# Injury characteristics and outcome of road traffic accident among victims at Adult Emergency Department of Tikur Anbessa specialized hospital, Addis Ababa, Ethiopia: a prospective hospital based study

**DOI:** 10.1186/s12873-015-0035-4

**Published:** 2015-05-20

**Authors:** Mohammed Seid, Aklilu Azazh, Fikre Enquselassie, Engida Yisma

**Affiliations:** Department of Nursing, Collage of Health Sciences, Wollo University, Dessie, Ethiopia; Department of Emergency Medicine, School of Medicine, College of Health Sciences, Addis Ababa University, Addis Ababa, Ethiopia; School of Public Health, College of Health Sciences, Addis Ababa University, Addis Ababa, Ethiopia; Department of Nursing and Midwifery, School of Allied Health Sciences, College of Health Sciences, Addis Ababa University, Addis Ababa, Ethiopia

**Keywords:** Road traffic accident, Injury characteristics, Outcome, Tikur Anbessa specialized hospital, Addis Ababa, Ethiopia

## Abstract

**Background:**

Road traffic injuries are the eighth leading cause of death globally, and the leading cause of death for young people. More than a million people die each year on the world’s roads, and the risk of dying as a result of a road traffic injury is highest in Africa.

**Methods:**

A prospective hospital based study was undertaken to assess injury characteristics and outcome of road traffic accident among victims at Adult Emergency Department of Tikur Anbessa specialized hospital, Addis Ababa, Ethiopia. A structured pre-tested questionnaire was used to gather the required data. The collected data were analyzed using SPSS version 20.0. Hierarchical multiple regression analysis was used to identify predictors of fatalities among the road traffic crash victims.

**Results:**

A total of 230 road traffic accident victims were studied. The majority of the study subjects were men 165 (71.7 %) and the male/female ratio was 2.6:1. The victims’ ages ranged from 14 to 80 years with the mean and standard deviations of 32.15 and ± 14.38 years respectively. Daily laborers (95 (41.3 %)) and students (28 (12.2 %)) were the majority of road traffic accident victims. Head (50.4 %) and musculoskeletal (extremities) (47.0 %) were the most common body region injured. Fractures (78.0 %) and open wounds (56.5 %) were the most common type of injuries sustained. The overall length of hospital stay (LOS) ranged from 1 day to 61 days with mean (± standard deviation) of 7.12 ± 10.5 days and the mortality rate was 7.4 %. Hierarchical multiple regression analysis showed that age of the victims (ß = 0.16, p < 0.05), systolic blood pressure on admission (ß = −0.35, p < 0.001) and Glasgow coma scale (ß = −0.44, p < 0.001) were statistically significant predictors of fatalities among the victims.

**Conclusions:**

This study showed diverse injury characteristics and high morbidity and mortality among the victims attending Adult Emergency Department of Tikur Anbessa specialized hospital, Addis Ababa, Ethiopia. The findings reflect that road traffic accident is a major public health problem. Urgent road traffic accident preventive measures and prompt treatment of the victims are warranted in order to reduce morbidity and mortality among the victims.

**Electronic supplementary material:**

The online version of this article (doi:10.1186/s12873-015-0035-4) contains supplementary material, which is available to authorized users.

## Background

Approximately 1.24 million people die every year on the world’s roads, and another 20 to 50 million sustain nonfatal injuries as a result of road traffic crashes [1]. Road traffic injuries are the eighth leading cause of death globally, and the leading cause of death for young people aged 15–29 [2]. Almost 60 % of road traffic deaths are among 15–44 year olds worldwide and for every road traffic fatality, only at least 20 people sustain non-fatal injuries [1, 3].

The overall global road traffic fatality rate is 18 per 100 000 population among which middle-income countries have the highest annual road traffic fatality rates, at 20.1 per 100 000 [1]. Half of the world’s road traffic deaths occur among motorcyclists (23 %), pedestrians (22 %) and cyclists (5 %) – i.e. “vulnerable road users” – with 31 % of deaths among car occupants and the remaining 19 % among unspecified road users [1]. Motor vehicle accidents are the leading cause of death in adolescents and young adults worldwide [4].

Eighty percent of road traffic deaths occur in middle-income countries, which account for 72 % of the world’s population. The risk of dying as a result of a road traffic injury is highest in Africa (24.1 per 100 000 population) and 38 % of all African road traffic deaths occur among pedestrians [1].

The injury characteristics for road traffic accident in developing countries differ from developed countries. Pedestrians are most vulnerable to injury and death due to a number of factors, including poor knowledge and practice of road safety measures by the general population, recklessness behavior of motorists, and high speed driving.

A study done in Tanzania did report that students and businessmen are the largest groups of road traffic crash victims and limb and head injuries are the most common types of injury sustained which predisposed victims to prolonged hospitalization and mortality [5]. A study conducted in Addis Ababa, Ethiopia on adult limb fractures in Tikur Anbessa hospital caused by road traffic injuries showed that road traffic injuries were responsible for almost half of the musculoskeletal injuries [6].

A significant proportion of patients who sustain a road traffic injury incur permanent disability, through amputation, head injury or spinal cord injury [1]. However, data on the number of people who incur a permanent disability as a result of road traffic accidents is not well documented in developing countries like Ethiopia. A study conducted by capture-recapture method from June 2012 to May 2013, on one of the busiest highways of Ethiopia, the Addis Ababa – Hawassa highway showed that deaths and injuries among females, younger age victims, cyclists/motorcyclists and pedestrians were underreported by traffic police [7]. The availability and efficiency of an adequate pre-hospital care system in Ethiopia is very limited though access to pre-hospital services and quick evacuation and transport to hospital can save many lives, since the majority of those who die do so before they reach a hospital [1].

Few research has been conducted in Ethiopia regarding road traffic accidents. There was no similar study conducted in Tikur Anbessa specialized hospital to the best of our knowledge. Therefore, the aim of this study was to assess injury characteristics and outcome of road traffic accident among victims at Adult Emergency Department of Tikur Anbessa specialized hospital, Addis Ababa, Ethiopia.

## Methods

### Study setting and period

The study was conducted between January and March 2013 in Tikur Anbessa specialized hospital in Addis Ababa, the capital city of Ethiopia and seat of African Union and United Nations World Economic Commission for Africa. Addis Ababa has a total population of 2,738,248; of whom 1,304,518 are men and 1,433,730 are women [8]. Tikur Anbessa specialized hospital is the largest referral, tertiary care hospital in Ethiopia and teaching hospital for Addis Ababa University, College of Health Sciences, Addis Ababa, Ethiopia since 1998. The hospital has 1262 various rooms and has been providing organized emergency care services [9].

### Study design

A prospective hospital based study was used to assess the injury characteristics and outcome of road traffic accident among victims at the Adult Emergency Department of Tikur Anbessa specialized hospital, Addis Ababa, Ethiopia.

### Study subjects and inclusion and exclusion criteria

The source population comprised of all patients attending the Adult Emergency Department of Tikur Anbessa specialized hospital. The study subjects were all road traffic accident victims of all gender and aged 14 years and above attending the Adult Emergency Department of the hospital between January and March 2013 irrespective of injury severity and who consented for the study. Patients who were unsuccessful to give proper information and those who had no accompanying relative or informant to consent for the study were excluded from the study. Recruitment of patients to participate in the study was done at the Adult Emergency Department of the hospital. Patients were screened for inclusion criteria described above and enrolled consecutively into the study. Accordingly, 230 road traffic accident victims who were consecutively enrolled at Adult Emergency Department were followed up till they were discharged or died. And information was collected using a pre-tested structured questionnaire and reviewing of the victims’ medical records (see Additional file 1).

### Data collection

A pre-tested and structured, interviewer administered questionnaire was adapted from previous studies [4, 6] and reviewing relevant literatures to the problem under the study to include all the possible variables that address the objective of the study. The questionnaire was designed to obtain information on variables included in the study such as socio-demographic profile (age, sex and occupation), mechanism of injury, type of road users (pedestrians, drivers, passengers) and date and time at which the accident occurred. Additionally, medical records of the victims were also reviewed to obtain information regarding body region injured, types of injury, procedures performed and the outcome of the victims (length of hospital stay, mortality and disability). The severity of injury was determined using the Kampala trauma score II (KTS II) adopted from earlier study [10]. The KTS II descriptions are displayed in Table 1. Patients with head injuries were classified according to Glasgow Coma Scale (GCS) into: severe (GCS 3–8), moderate (GCS 9–13) and mild (GCS 14–15) [11].

**Table 1:** Description of Kampala Trauma Score (KTS II)

	Description	Score
A	Age (in years)	
	5–55	1
	<5 or > 55	0
B	Systolic Blood Pressure on admission	
	More than 89 mm Hg	2
	Between 89–50 mm Hg	1
	Equal or below 49 mm Hg	0
C	Respiratory rate on admission	
	0-29/minute	2
	30+	1
	≤9/minutes	0
D	Neurological status	
	Alert	3
	Responds to verbal stimuli	2
	Responds to painful stimuli	1
	Unresponsive	0
E	Score for serious injuries	
	None	2
	One injury	1
	More than one	0

Kampala Trauma Score total = A + B + C + D + E; Scores, 9– 10: Mild injury; 7– 8: Moderate injury; 6 or less (≤6): Severe injury [10]

### Data analysis

Data entry was performed using the software Epi Info version 3.5.1. Data cleaning was done via a record screen of Epi Info using the list command and the sort button and by cross-checking with the hard-copy questionnaire. The data were then exported to SPSS version 20 for further analysis. Frequency distributions, cross-tabulations and graphs were used to describe the variables of the study. Hierarchical multiple regression analysis was performed to investigate the ability of variables such as vehicle type, crash type and road user type to predict the fatalities among victims, after controlling for other predictor variables such as age, Glasgow coma scale, Systolic blood pressure at admission (SBP) and severity of the trauma. Observed differences between each step of the hierarchical multiple regression model and predictor variables were considered statistically significant at P < 0.001 and P < 0.05.

### Ethical considerations

Ethical clearance was obtained from the ethical review committee of the Department of Emergency Medicine of Addis Ababa University. An informed written consent was sought from patients or relatives and protection of the rights of the study participants was ensured by giving them due freedom to participate in the study or not to participate. Privacy and confidentiality were maintained during the interview.

## Results

### Socio-demographic characteristics of the study subjects

Out of 690 trauma patients who visited Adult Emergency Department of Tikur Anbessa specialized hospital between January and March 2013, two hundred and fifty (36.23 %) of the patients were road traffic accident victims among which 230 road traffic accident victims were enrolled and studied during the period under the study. The study participants comprised of 165 (71.7 %) men and 65 (28.3 %) women, resulting in a male to female ratio of 2.6:1. The patients’ ages ranged from 14 to 80 years with the mean and standard deviations of 32.15 and ± 14.38 years respectively. The median and the mode were 26 and 25 years respectively. The modal age group was 14–25 years, accounting for 107 (46.5 %) patients. The majority of the road traffic victims were daily laborers (95 (41.3 %)) followed by students (28 (12.2 %)). Regarding educational status of road traffic accident victims, 165 (71.74 %) of them had attended formal school of which 59 (35.76 %) and 73 (44.24 %) of the victims had a primary and secondary school education respectively while 33 (20.00 %) of the victims had reported higher educational level education (see Table 2).

**Table 2:** Socio-demographic characteristics of road traffic accident victims at Adult Emergency Department of Tikur Anbessa specialized hospital, Addis Ababa, Ethiopia, January—March 2013

Variables	Frequency (*N* = 230)	Percentage
Age		
14–25	107	46.5
26–35	54	23.5
36–45	28	12.2
45^+^	41	17.8
Sex		
Male	165	71.7
Female	65	28.3
Religion		
Orthodox	160	69.6
Muslim	46	20.0
Protestant	24	10.4
Ethnicity		
Oromo	95	41.3
Amhara	85	37.0
SNNPR	27	11.7
Tigray	18	7.8
Others	5	2.2
Educational status		
Cannot read and write	45	19.6
Can read and write	20	8.7
Primary school	59	25.7
Secondary school	73	31.7
Higher education	33	14.3
Marital status		
Single	114	49.6
Married	90	39.1
Divorced	11	4.8
Widowed	15	6.5
Occupation		
Daily laborer	95	41.3
Student or trainee	28	12.2
Government employee	23	10.0
Car driver	18	7.8
Businessman (Merchant)	18	7.8
Farmer	18	7.8
House wife	15	6.5
Others	15	6.5

### Place, time and circumstances of road traffic accident injuries

Out of 230 road traffic accidents that occurred during the period under the study, 139 (60.4 %) of those accidents occurred in Addis Ababa city while 91 (39.6 %) occurred out of the city. Lideta, Kolefe-keranio, Nifas-silk-lafto and Kirkos sub-cities were the places where 61.5 % of the accidents occurred in the city whereas Deberzeit (Harar Ber) and Ambo Ber roads accounted for about 60 % of the road traffic accidents that occurred out of Addis Ababa city.

The majority 156 (67.9 %) of road traffic accidents occurred during the day time while only 74 (32.1 %) occurred during the night time. The incidence of the accident was higher in the afternoon (6:00–12:00 PM) 91 (39.6 %) compared to occurrence of the accident from early morning to mid-day (65 (28.3 %)). On contrary, more road traffic accidents were occurred from evening up to mid-night 64 (27.8 %) than after mid-night to early morning time period 10 (4.3 %). Moreover, most of the road traffic accidents occurred on Sundays 41 (17.8 %) followed by Saturdays 37(16.1 %). The occurrence of road traffic accidents during the working days (Monday to Friday) ranged from 24—36 (10.4—15.7 %).

**Fig. 1:**
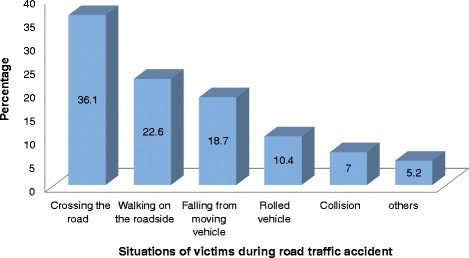
Situations of victims during road traffic accident, January—March 2013

Regarding the conditions of the victims during the occurrence of the accidents, 83 (36.1 %) victims were crossing the road, 52 (22.6 %) and 43 (18.7 %) of the victims were injured while walking on the roadside and rolled vehicle respectively while those victims who reported falling from a moving vehicle, collisions with other vehicle and other situations accounted for about 52 (22.6 %) of the accidents (See Fig. 1).

Long-distance travelling Minibuses (38 (16.5 %)) were responsible for the majority of road traffic crashes, followed by Taxis, Heavy good vehicles, Long-distance travelling Bus, Isuzu and other means of transportation (private Automobile, Pickup trucks, Motorcycle and others) in only 0.2 % of cases (Table 3). Moreover, city Taxi (17 (20.4 %)), private Automobiles (13 (15.6 %)), long-distance traveling Minibuses (11 (13.2 %)) and Isuzu (10 (12.0 %)) caused injuries while the victims were crossing the road while more than half of roiled vehicle mechanism of accidents were caused by long-distance traveling Minibuses (15 (48.3 %)) and heavy good vehicles (10 (23.2 %)). Pedestrians (144 (62.6 %)) accounted for the majority of the victims, followed by passengers (56 (24.3 %)), drivers (15 (6.5 %)), motorcyclists (5 (2.2 %)), cyclists (3 (1.3 %)) and others (7 (3.0 %)).

**Table 3:** Type of vehicles involved in causing injuries among the victims, January—March 2013

Type of vehicles	Frequency (*N* = 230)	Percentage
Long-distance travelling Minibus	38	16.5
Taxi	34	14.8
Heavy good vehicles	32	13.9
Long-distance travelling Bus	25	10.9
Isuzu	22	9.6
Private automobile	19	8.3
Pickup trucks	17	7.4
Lada	12	5.2
Motorcycle	11	4.8
Bajaj	9	3.9
Other vehicle types	11	4.8

The majority of victims (200 (86.96 %)) reported to the Adult Emergency Department within 24 hours after the injury, of which 62 (27.0 %) of the victims were arrived within the golden hour (within the first hour) of the trauma. None of the patients received any pre-hospital care and only 52 (22.61 %) and 9 (3.9 %) of the victims were brought in by ambulance and police car to hospital respectively.

### Injury characteristics

Head and the musculoskeletal (extremities) were the most common body region injured accounting for 50.4 % and 47.0 % of cases respectively (Table 4).

**Table 4:** Site of injuries among the road traffic accident victims at Adult Emergency Department of Tikur Anbessa specialized hospital, Addis Ababa, Ethiopia, January—March 2013

Site of injury	Frequency	Percentage
Head	116	50.4
Musculoskeletal	108	47.0
Spine	33	14.3
Chest	32	13.9
Maxillofacial	15	6.5
Pelvis	13	5.7
Abdomen	12	5.2

According to Kampala Trauma Score II (KTS II) classification of trauma severity, more than half of the victims 119 (51.74 %) encountered mild trauma (KTS II = 9–10) while moderate injuries (KTS II = 7-8) and severe injuries (KTS II ≤ 6) were recorded in 86 (37.39 %) and 25 (10.87 %) of the victims respectively. The fatality rate of mild, moderate and severe trauma were 0 % (no death), 35.29 % (6 deaths) and 64.71 % (11 deaths) respectively.

The Glasgow coma scale score of 116 (50.4 %) in head injury victims were mild in 65 (56.03 %), moderate in 23 (19.83 %) and severe in 28 (24.14 %) of cases respectively. Moreover, the mortality rate for severe head injury was 11 (73.3 %), 3 (20.0 %) for moderate and 1 (6.7 %) for mild head injuries.

Out of the total 230 road traffic accident victims, open wound was sustained by 130 (56.5 %) of victims while dislocation was sustained only by 12 (5.2 %) of the victims. Different type of fractures were sustained by 177 (78.0 %) of the victims of which the majority of the fracture injuries sustained were accounted for lower limb fracture (64 (36.2 %) while pelvic fracture injuries (9 (5.1 %)) accounted for the least fracture type sustained. Thoracic injuries were also sustained by 19 (8.3 %) of the road traffic accident victims of which pneumohemothorax (10 (52.6 %)) was the leading types of the thoracic injuries followed by hemothorax and pneumothorax accounting for 10.5 % each. Contusion constituted for the majority of intracranial bleeding in 27 (54.0 %) of the cases while subarachnoid bleeding sustained by only 4 (5.8 %) of the victims accounting for the least intracranial hemorrhage accident. Moreover, only 7 (3.0 %) visceral injuries were sustained by the victims (see Table 5).

**Table 5:** Type of injuries sustained among the road traffic accident victims, January—March 2013

Types of injury	Frequency of injuries	Percentage
Open wounds	130	56.5
Fractures	177	78.0
Lower limb fractures	64	36.2
Skull/maxillofacial fractures	33	18.6
Spinal fractures	20	11.3
Upper limb fractures	19	10.7
Rib fracture	17	9.6
Clavicle fractures	15	8.5
Pelvic fractures	9	5.1
Thoracic injuries	19	8.3
Pneumohemothorax	10	52.6
Hemothorax	2	10.5
Pneumothorax	2	10.5
Emphysema	2	10.5
Pneumomedaistinitis	1	5.2
Diaphragmatic injury	1	5.2
Pleural effusion	1	5.2
Intracranial hemorrhage	69	30
Contusion	27	39.1
Subdural	11	15.9
Intracerebral	11	15.9
Epidural	10	14.5
Pheumocephalus	6	8.7
Subarachnoid	4	5.8
Visceral injuries	7	3.0

### Treatment of the road traffic victims

Of the total 187 procedures performed, treatment of fracture (108 (57.7 %)) and wound debridement (27 (14.4 %)) were more frequently procedures performed. Fifteen (8.0 %) under water seal drainage was performed for victims with thoracic injuries. Moreover, different surgical producers such as craniotomy, Burr hole, exploratory laparotomy, skin grafting, tracheotomy, limb re-amputation and ventricular shunt were also performed (See Table 6 below).

**Table 6:** Types of surgical procedures performed for the victims at Adult Emergency Department of Tikur Anbessa specialized hospital, Addis Ababa, Ethiopia, January—March 2013

Types of surgical procedure	Frequency (*N* = 187)	Percentage
Treatment of fracture	108	57.7
Wound debridement	27	14.4
Under water seal drainage	15	8.0
Relocation of dislocation	8	4.3
Craniotomy	5	2.7
Exploratory laparatomy	5	2.7
Burr hole	5	2.7
Skin grafting	4	2.1
Tracheostomy	2	1.1
Limb re-amputation	2	1.1
Ventricular shunt	1	0.5
Other surgical procedures	5	2.7

### Clinical outcomes of road traffic accident victims

**Fig. 2:**
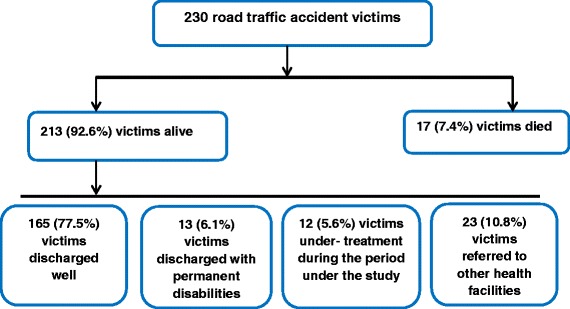
Flow chart showing the clinical outcome of road traffic accident victims who visited Adult Emergency Department of Tikur Anbessa specialized hospital, Addis Ababa, Ethiopia, January—March 2013

Out of the 230 victims studied, 213 (92.6 %) victims were alive while 17 (7.4 %) of them were died during the course of the treatment. Among the victims that were alive, 165 (77.5 %) patients were discharged well without permanent disability, 13 (6.1 %) patients were discharged with permanent disabilities such as severe spinal cord injury, 23 (10.8 %) of the victims were referred to other health facilities for different reasons and the rest 12 (5.6 %) of the victims were under treatment till the end of the study period (Fig. 2).

The overall length of hospital stay (LOS) ranged from 1 day to 61 days with the mean (± standard deviation) of 7.12 ± 10.5 days. The median was 3 days.

### Hierarchical multiple regression analysis of variables predicting fatalities among victims

Table 7 below shows hierarchical multiple regression analysis performed to investigate the ability of vehicle type, crash type and road user type to explain/predict fatalities among victims of road traffic accidents, after controlling for others predictors such as age, Glasgow coma scale, SBP at admission and the severity of trauma.

In the first step of hierarchical multiple regression, four predictors were entered: age, Glasgow coma scale, SBP at admission and severity of trauma. This model was statistically significant F (4, 225) = 34.45; p < 0.001 and explained 38.0 % of variance in fatalities among victims of the accident. After entry of vehicle type, crash type and road user type at Step 2 the total variance explained by the model as a whole was 38.6 % (F (7, 222) = 19.92; p < 0.001). The introduction of vehicle type, crash type and road user group type explained only additional 0.6 % variance in fatalities among the victims, after controlling for age, Glasgow coma scale, SBP at admission and the severity of trauma (R^2^ Change = 0.006; F (3, 222) = 0.74; p > 0.05). This was not showed statistically significant change. In the final model three out of six predictor variables were statistically significant, with age of the victims recording a higher Beta value (ß = 0.16, p < 0.05) than the SBP at admission (ß = −0.35, p < 0.001) and Glasgow coma scale (ß = −0.44, p < 0.001) (Table 7).

**Table 7:** Hierarchical multiple regression model showing fatalities from road traffic accidents

Variables entered into the model	R	R^2^	R^2^ change	*B*	SE	*ß*	t	*P*
Step 1	0.62	0.38**						
Age				0.14	0.05	0.16	2.83	0.005*
Glasgow coma scale				−0.17	0.02	−0.44	−7.53	0.000**
SBP at admission				−0.25	0.04	−0.34	−6.57	0.000**
Severity of trauma				0.01	0.03	0.01	0.22	0.829
Step 2	0.62	0.386**	0.006					
Vehicle type				0.001	0.17	0.002	0.04	0.968
Crash type				0.03	0.02	0.09	1.47	0.144
Road user type				−0.02	0.03	−0.06	−0.89	0.377

Statistical significance: *p < 0.05; **p < 0.001

***R*** ^***2***^ amount of variance explained by independent variables**,** ***R*** ^***2***^ ***change*** Additional variance in dependent variable, ***B*** Unstandardized coefficient, ***ß*** Standardized coefficient, ***SE*** Standard Error, ***t*** estimated coefficient (B) divided by its own SE

## Discussion

This study focused on injury characteristics and outcome of road traffic accident among victims attending Tikur Anbessa specialized hospital, Addis Ababa, Ethiopia, specifically, at Adult Emergency Department. Findings from this study may therefore be regarded as a window that provides a prevue into current situation of road traffic accident and its outcome among the victims in the study area.

In this study, the majority of road traffic accident victims were young in their most reproductive and productive years and showed male dominance over women. The male dominance in the present study is consistent with earlier study findings reported in different places [5, 12–17]. Reproductive and productive age group represents the economically active age and the reason for their high incidence of road traffic crash may reflects their high economic activity levels and participation in high-risk activities such as recklessness driving/riding, over-speeding, driving/riding under the influence of alcohol and drugs and driving/riding without wearing any protective mechanisms [5]. Male predominance in this study is due to their increased participation in high-risk activities and this may demands an urgent public policy response.

In present study, the majority of the road traffic victims were daily laborers (41.3 %) followed by students (12.2 %). This result implies that students and daily laborers were injured because of the fact that this group of people haste through heavy traffic to the school and get to their jobs and this finding is consistent with previous studies by others [5, 13, 15]. Students are usually involved in road traffic accidents as they go through heavy traffic to and from their schools while daily laborers are often involved while performing their day to day activities which necessitate movement from one place to another in order to get their businesses.

In this study, Long-distance travelling Minibuses were responsible for the majority (16.5 %) of road traffic crashes, followed by city Taxi and Heavy good vehicles. The prevalence of motorcycle injuries in this study is very low which accounted only for 4.8 % of the cases. This may indicate that in Addis Ababa city and other parts of the country people may not use motorcycle more frequently as means of transportation. However, this finding is in contrary to two study findings reported from in Tanzania where the motorcycle was responsible for the majority of road traffic crashes [5, 15]. Long-distance travelling Minibuses use is mostly used by people in Ethiopia as it is selected because of its accessibility and faster means of transportation in most cities. However their use may be characterized by non-seat belt use by passengers, passenger overload, lack of certified driver training and valid licensing, over speed and reckless driving behavior, and possible use of alcohol and drugs.

Pedestrians (62.6 %) accounted for the majority of road traffic accident victims in our study and this finding is consistent with other earlier studies [4, 5, 13, 18] and it is in contrast to other studies which reported passengers as the majority of cases [15, 19]. High incidence of pedestrians in the present study may reflect low community awareness on road use and therefore education on how to use roads should be warranted.

The majority 156 (67.9 %) of road traffic accidents occurred during the day time in the present study and this finding is consistent with the findings reported from studies elsewhere [5, 13, 15, 20]. This may justify the fact that during the day time, there are increased human activities as well as increased traffic overcrowding in cities which is responsible for causing road traffic accident injuries. Knowing the time of injury in road traffic accident victims is important for prevention strategies.

The pre-hospital care of trauma patient has been reported to be the most important factor in determining the ultimate outcome after the injury [15]. None of road traffic accident victims had pre-hospital care and only (22.61 %) of the victims were brought in by ambulance to hospital in the present study. The finding is however, higher than study finding reported in Tanzania [5] where only 0.8 % of road traffic crash victims were brought in by ambulance. Though there is difference between these two countries, the finding may indicates that the majority of the victims were brought in by relatives and police who are not trained on how to take care of these patients during transportation. This necessitate good access to pre-hospital services and quick transportation system to hospital in order to save lives, since the majority of those who die do so before they reach a hospital.

Head and the musculoskeletal (extremities) were the most common body region injured in the present study accounting for 50.4 % and 47.0 % of cases respectively. This finding is consistent with previous studies [5, 12, 13, 15, 16]. The high figure of musculoskeletal injuries may attributable to the large number of pedestrians in current study. Pedestrians are unprotected road users and therefore they are highly exposed to high risk of extremity (limb) injuries [21]. Enforcement of helmet use by motorcyclists and cyclists might be helpful and may decrease head injuries.

Most of victims in present study were treated surgically, which is in agreement with other similar studies [5, 13, 15, 16]. The high incidence of surgical treatment in our study is attributable to the high incidence of road traffic accident victims with moderate to severe injuries which required surgical intervention.

In the current study, (92.6 %) of victims were alive while (7.4 %) of them were died during the course of the treatment. This figure is however, lower than from that reported in Tanzania [5] where the mortality rate was 17.5 %. High mortality rate in the present study was recorded in patients with severe trauma, admission SBP **>**89 mmHg, severe head injury and in the victims with age group of 14–55 years of age than >55 years of age group. And according to Kampala Trauma Score II classification of trauma severity, more than half of the victims (51.74 %) encountered mild trauma (KTS II = 9–10) while moderate injuries (KTS II = 7-8) and severe injuries (KTS II ≤ 6) were recorded in (37.39 %) and (10.87 %) of the victims and the fatality rate of mild, moderate and severe trauma were 0 % (no death), 35.29 % (6 deaths) and 64.71 % (11 deaths) respectively in current study. The low morality rate seen in present study is may due to the small size of the study subjects (230 road traffic accident victims) at Adult Emergency Department of Tikur Anbessa hospital, Addis Ababa, Ethiopia compared to 1678 road traffic crash victims at at Bugando Medical Centre in Northwestern Tanzania [5].

The length of hospital stay (LOS) has been reported to be an important measure of morbidity among trauma patients [5]. Prolonged hospitalization is associated with an unacceptable burden on resources for health and undermines the productive capacity of the population through time lost during hospitalization and disability [5]. In this study, overall mean LOS was lower than from that reported by others [5, 12, 15]. Prolonged LOS in our study is attributable to presence of large number of patients with bone fractures (78.0 %) which took time to heal.

The limitations of this study could include the following. Firstly, as this study is confined to only Tikur Anbessa specialized hospital, Addis Ababa, Ethiopia, the findings may not be generalizable to other hospitals in Addis Ababa city and out of Addis Ababa. The other limitation of the study could be the small sample size which may make estimates unstable and associations between dependent and independent variables undetectable**.**

## Conclusions

This study showed diverse injury characteristics and high morbidity and mortality among the victims attending Adult Emergency Department of Tikur Anbesa specialized hospital, Addis Ababa, Ethiopia. The findings reflect that road traffic accident is a major public health problem. The young adult male in their economically productive age-group were mostly involved. Daily laborers and students were the largest groups of road traffic accident victims. Head and musculoskeletal injuries were the most common types of injury sustained. And of the 230 victims studied, 213 (92.6 %) victims were alive while 17 (7.4 %) of them were died.

Based on the findings of this study, urgent road traffic accident preventive measures and prompt treatment of the victims are warranted in order to reduce morbidity and mortality and/or bad outcome among the victims. Strengthening and enforcement of safety rules will help in reducing the occurrence of road traffic accident. Awareness creation educations regarding safety rules for young adult males, students and businessmen is also essential in order to reduce the road traffic accident and its outcome.

## Additional file

Additional file 1:
**Structured questionnaire on Injury characteristics and outcome of road traffic accidents among victims.**

## Abbreviations

CI: Confidence interval
GCS: Glasgow coma scale
KTS: Kampala trauma scale
LOS: Length of hospital stay
