# Supplementary material for: Injury characteristics and outcome of road traffic accident among victims at Adult Emergency Department of Tikur Anbessa specialized hospital, Addis Ababa, Ethiopia: a prospective hospital based study
Source: BMC Emerg Med. 2015 May 20;15:10. doi: 10.1186/s12873-015-0035-4 (PMC4493961; doi:10.1186/s12873-015-0035-4)
Supplement: Additional file 1: — Structured questionnaire on Injury characteristics and outcome of road traffic accidents among victims. [file 12873_2015_35_MOESM1_ESM.pdf]

ADDIS ABABA UNIVERSITY, COLLEGE OF HEALTH SCIENCE, SCHOOL OF MEDICINE, DEPARTMENT OF EMERGENCY MEDICINE

Consent Form

This questionnaire is prepared to assess the injury characteristics and outcome of road traffic accident among victims at Adult Emergency Department of Tikur Anbesa specialized hospital, Addis Ababa, Ethiopia.

Consent form and introduction

My name is ______________________________. I am working with Mr. --------- who is doing a research on the injury characteristics and outcome of road traffic accident among victims. We are interviewing victims and/or relatives to assess the injury characteristics and outcome of road traffic accident among victims. I am going to ask you some questions that could be important for organizations working on road safety and road traffic accident preventions. Your name will not be written in this form and the information you give will be kept confidential. If you don't want to answer all of or some of the questions, you do have the right to do so. However your willingness to answer all of the questions would be appreciated.

Would you participate in responding to the questions in this questionnaire?

Yes ☐ No ☐

If the answer is "**Yes**", thank and conduct the interview.

If the answer is "**No**", thank and transfer to other respondent.

Sign of interviewee: ------------------------------------

Date of interview: ---

Name and sign of interviewer: ---

Name of the supervisor --------------------------------- signature ------------

Date of checking---

Remark: 1. Complete 2. Incomplete

Questionnaire

Interviewer-administered questioner and record review checklist for the assessment of the injury characteristics and outcome of road traffic accident among victims at Adult Emergency Department of Tikur Anbesa specialized hospital, Addis Ababa, Ethiopia.

Name of the hospital: ---

Name of the reviewer: --

Date: ---

Day: ---

Card number: ---------------------------------

Part I - Socio demographic characteristics

**Questions**		**Responses**
1	Sex	Male------------------------------1<br>Female --------------------------2
**2**	Age (in years)	specify--------------------------
**3**	Religion	Orthodox ------------------------1<br>Catholic ---------------------------2<br>Protestant--------------------------3<br>Muslim -------------------------4<br>Others (specify)-------------------5
4	Ethnicity	Specify -----------------------------
5	Educational status	Illiterate Cannot read and Write ----1<br>Can read and Write--------------------2<br>Primary school-------------------------3<br>Secondary school-----------------------4

		Higher education ----------------------5
6	Marital status	Married---------------------------------1<br>Single ---------------------------------2<br>Divorced-------------------------------3<br>Widowed------------------------------4
**7**	Occupation	Working (specify) -------------------1<br>Off sick ------------------------------2<br>Student/trainee ----------------------3<br>Out of work ------------------------- 4<br>Housekeeper -----------------------5<br>Retired -------------------------------6<br>businessmen ------------------------7<br>Others (specify)----------------------8

Part II -The following questions are regarding the pattern of injury among victims

**Questions**		**Responses**
8	Geographical location of the accident	Specify ---------------------------------
9	Was there any death at the scene?	Yes ---------------------------------1<br>No -----------------------------------2
10	If yes for “Q.No. 10” how many?	Specify ---------------------------------
11	Which one was your role during the accident?	Vehicle driver -------------------1<br>Pedestrian--------------------------2<br>Motorcyclist ----------------------3<br>Cyclist -----------------------------4<br>Vehicle passenger ----------------5<br>Others (specify) -------------------6

12	Situation of the victim during RTA injury	Waking on the road side -----------------1<br><br>Fall from a moving vehicle --------------2<br><br>Rolled vehicle------------------------------3<br><br>Collision-------------------------------------4<br><br>Others--5
13	Which vehicles type caused the injury?	Taxi----------------------------------1<br><br>Motorcycle---------------------------2<br><br>Bicycle-------------------------------3<br><br>Bus/minibus-------------------------4<br><br>Heavy goods vehicles--------------5<br><br>Pick-up trucks-----------------------6<br><br>Others---------------------------------7
14	Date of accident in Ethiopian calendar (e.g. January, 6 /2005)	Specify --------------------------------
15	Day of accident (e.g. Monday)	Specify --------------------------------
16	Time of the occurrence of the accident in Ethiopian calendar	Morning to mid-day (after 12-6 AM)--------------1<br><br>Afternoon (after 6-12 PM)---------------------------2<br><br>Up to midnight(after 12-6 PM)----------------------3<br><br>After midnight to morning (after 6 to 12 AM)----4
17	Time taken to reach to the first heath facility after accident	Specify --------------------------------
18	Time taken to reach to TikurAnbesa Specialized Hospital	Specify --------------------------------
19	Means of transportation to the hospital	Ambulance-----------------------------1<br><br>Taxi-------------------------------------2<br><br>Private car-------------------------------3

		Carried------------------------------------4<br>Others specify ----------------------------5
20	Is there any treatment given?	Yes --1<br>No---2
21	If yes for “Q No 20” where did you received the treatment?	At the scene--------------------------------1<br>In the health center -----------------------2<br>In the hospital ---------------------------3<br>Others specify ----------------------------4
22	Which Regions(s) of the body were injured?	Head --------------------------------------1<br>Maxillofacial ------------------------------2<br>Chest---------------------------------------3<br>Abdomen-----------------------------------4<br>Pelvis --------------------------------------5<br>Spines -------------------------------------6<br>Musculoskeletal (extremities) -----------7
23	Glasgow Coma Scale (GCS) score at admission for head injury victims	3-8---------------------------------------1<br>9-13------------------------------------ -2<br>14-15------------------------------------3
24	Systolic Blood Pressure at the admission	Specify --------------------------------------
25	Respiratory rate at admission	Specify----------------------------------
26	Neurological status	Alert--1<br>Responds to verbal stimuli---------------2<br>Responds to painful stimuli--------------3<br>Unresponsive------------------------------4
27	Presence of open wound	Yes ----------------------------------1<br>No -----------------------------------2

28	Type of fractures suffered by the victims	Skull/maxillofacial fractures -----------1<br>Clavicle fractures------------------------2<br>Spinal fractures---------------------------3<br>Rib fractures-----------------------------4<br>Pelvic fractures---------------------------5<br>Upper limb fractures---------------------6<br>Lower limb fractures--------------------7
29	Types of hemorrhages the patient suffered?	Subdural ---------------------------1<br>Epidural -----------------------------2<br>Subarachnoid ----------------------3<br>Intracerebral------------------------ 4
30	Type of visceral injuries the patient suffered?	Spleen--1<br>Intestines-------------------------------------2<br>Liver--3<br>Urinary bladder-----------------------------4<br>Kidney--------------------------------------5<br>Others(specify)-----------------------------6
31	Type of chest injury the patient suffered?	Pneumothorax----------------------------1<br>Hemothorax------------------------------2<br>Pneumohemothorax----------------------3<br>Rib fracture-------------------------------4<br>Cardiac temponade ----------------------5<br>Others (specify)-------------------------6
32	Had the victim suffered from traumatic limb amputation?	Yes (specify)--------------------------------1<br>No (specify)---------------------------------2

Part III -The following questions are regarding the outcome of injury among the victims

Questions		Responses
33	Length of hospital stay (per day)	Specify --
34	Which surgical procedures was performed for the victim?	Underwater seal drainage ----------------------1<br>Skin grafting--------------------------------------2<br>Limb re-amputation-----------------------------3<br>Treatment of fractures---------------------------4<br>Craniotomy/burr holes --------------------------5<br>Wound debridement ----------------------------6<br>Exploratory laparotomy-------------------------7<br>Other surgical procedures (specify)------------8
35	Type of permanent disabilities the patient suffered?	Traumatic limb amputations--------------------1<br>Permanent neurological deficit------------------2<br>Severe spinal injuries with paraplegia----------3<br>Post-traumatic seizures--------------------------4<br>Others (specify)------------------------------------5
36	Final Outcome	Discharged well without permanent disability----1<br>Discharged with permanent disabilities------------2<br>Died --3

Thank you!
